# Counterfeit ‘Xanax®’ tablets: A comparative study of clinical and seizure data in Victoria, Australia

**DOI:** 10.1111/add.70174

**Published:** 2025-08-26

**Authors:** Rebekka Syrjanen, Shaun L. Greene, Sarah E. Hodgson, Rachelle Abouchedid, Dimitri Gerostamoulos, Christie Magee, Melissa Bremner, Jennifer L. Schumann, Emerging Drugs Network of Australia – Victoria (EDNAV) research group

**Affiliations:** ^1^ Department of Forensic Medicine Monash University Southbank Victoria Australia; ^2^ Victorian Poisons Information Centre, Austin Health Austin Hospital Heidelberg Victoria Australia; ^3^ Emergency Department, Austin Health Austin Hospital Heidelberg Victoria Australia; ^4^ Department of Critical Care, Melbourne Medical School The University of Melbourne Parkville Victoria Australia; ^5^ Emergency Department, Bendigo Health Bendigo Hospital Bendigo Victoria Australia; ^6^ Toxicology Department Victorian Institute of Forensic Medicine Southbank Victoria Australia; ^7^ Victoria Police Forensic Services Department Drug Sciences Group Macleod Victoria Australia; ^8^ Monash Addiction Research Centre Monash University Frankston Victoria Australia

**Keywords:** alprazolam, benzodiazepine‐type, counterfeit alprazolam, new psychoactive substances, NPS, toxicosurveillance, Xanax®

## Abstract

**Background and aim:**

There is growing evidence of counterfeit benzodiazepine products containing other substances, including non‐regulated benzodiazepine‐type new psychoactive substances (NPSs). This study sought to compare detections of seized suspect counterfeit alprazolam products with clinical cases that reported use of an alprazolam‐containing product to better characterise community use.

**Design and setting:**

Observational study set in Victoria, Australia, using data from the Victoria Police Drug Sciences Group (which compiles information about seized drugs submitted for evidential analysis and intelligence purposes) and the Emerging Drugs Network of Australia – Victoria (EDNAV) project (a prospective, observational study collecting clinical and analytical data for illicit drug‐related presentations across a network of hospitals in Victoria, Australia).

**Cases:**

Police seizures expected to contain alprazolam (March 2020 and August 2022) and EDNAV cases with a reported exposure to an alprazolam‐containing product (September 2020 and August 2022).

**Measurements:**

Descriptive study outlining drug detections in seized tablets and blood samples from EDNAV cases, comparing patterns of detection and changes over time.

**Findings:**

A total of 623 police seizures were analysed, most commonly products labelled as ‘Xanax®’ (*n* = 266), ‘Kalma®’ (*n* = 196) or ‘Mylan®’ (*n* = 124). Thirty percent of seizures contained alprazolam only. A benzodiazepine‐type NPS was detected in 375 seizures (60.2%). Exposure to non‐prescribed alprazolam was reported in 11.2% (*n* = 125/1112) of EDNAV cases, with 68.8% identifying as male and a median age of 26 years (range 16–68 years). Eighty‐seven cases reported the use of ‘Xanax®’. Alprazolam was detected in 19 EDNAV cases. A benzodiazepine‐type NPS was detected in 78.4% of EDNAV cases. Both datasets saw a shift in detections from etizolam (2020) to clonazolam (2021) and then clobromazolam (2022).

**Conclusions:**

Suspect counterfeit alprazolam products seized by police in Victoria, Australia, in 2020 and 2022 commonly contained other drugs and/or new psychoactive substances, with an apparent limited consumer awareness of the tablet composition.

## INTRODUCTION

Counterfeit pharmaceuticals are a growing and profitable international industry, with global trade for these illicit products estimated at over $4.4 billion (USD) in 2016 alone [1]. In recent years, there has been growing evidence of counterfeit benzodiazepine products on the market that are substituted by or adulterated with other substances, such as non‐regulated benzodiazepine‐type new psychoactive substances (NPSs) [2, 3]. Benzodiazepine‐type NPSs are structural analogues of prescription benzodiazepines with similar sedative–hypnotic effects, but many have not been subject to the same rigorous pre‐clinical and clinical testing as their legitimate counterparts [4, 5]. Additional psychoactive substances have also been identified within these products, including fentanyl analogues and novel opioids [6, 7]. Concerningly, these products are often indistinguishable from their licit pharmaceutical counterparts, closely replicating product packaging and appearance [3].

Australia is one such jurisdiction that has noted the introduction of counterfeit benzodiazepine products in the illicit drug market [3]. Over the past 3 years, public health bodies from several Australian jurisdictions have released warnings about counterfeit alprazolam products, labelled ‘Kalma®’, ‘Mylan®’ and ‘Xanax®’, which have contained benzodiazepine‐type NPSs and/or other psychoactive substances [2, 3, 8]. Kalma® (manufactured by the Australian subsidiary of Mylan Pharmaceuticals) and Alprax® are currently registered pharmaceutical products in Australia. Xanax®‐branded alprazolam was withdrawn from the Australian market in 2015 following a cancellation request from the product sponsor Pfizer after alprazolam was rescheduled to a higher restriction level [9].

In Australia there does not appear to be the same level of consumer awareness in comparison with international jurisdictions, where people appear to be seeking specific benzodiazepine‐type NPSs [10, 11, 12, 13]. In a recent survey of people who use drugs in Australia, less than 1% of respondents who reported recent non‐prescribed benzodiazepine use reported the intentional use of a benzodiazepine‐type NPS [12]. This aligns with previous work from illicit drug toxicosurveillance within emergency departments, where none of the patients with an analytically confirmed exposure to a benzodiazepine‐type NPS reported their use by name [13]. Instead, almost half of the patients reporting use of a product presumed to be alprazolam (‘street’ alprazolam, ‘Mylan®’ or ‘Xanax®’) [13].

Toxicological data from clinical or coronial cases are limited in interpretation, with an inability to definitively ascribe the source of the drugs detected. Seizure data provide an insight into tablet composition but may not be reflective of products reaching the community. To better understand the use of these substances in the Victorian community, this descriptive study aims to compare police seizure data of suspect counterfeit alprazolam products with toxicological data from patients presenting to hospital following self‐reported ingestion of an alprazolam‐containing product between September 2020 and August 2022 in Victoria, Australia.

## METHODS

### Victorian drug seizure data

Drug seizure data were sourced from the Drug Sciences Group (DSG) database managed by the Victoria Police Chemical Drug Intelligence (CDI) Unit. Drug seizures include exhibits submitted to the Forensic Services Department for evidential analysis through the Drug Analysis Unit as well as exhibits submitted for analysis by and destruction through the CDI Unit for intelligence purposes. The submission of drug exhibits is influenced by court proceedings and the DSG submission criteria. A case generally includes a collection of items seized from one accused at one location; however, items may be seized from multiple offenders, and from different locations at different times, but belonging to the same investigation. For this study, a seizure is defined as a drug exhibit with a unique presentation and drug composition. For example, Kalma 2 mg tablets containing alprazolam and Kalma 2 mg tablets containing etizolam are considered two separate seizures, but they may originate from the same case.

This study was not pre‐registered within any research registry. The results from this study should be considered exploratory.

### Case selection and extracted variables

The DSG database was examined for case information related to tablet seizures that would be expected to contain alprazolam between March 2020 and August 2022. The tablet presentations included Alprax® (0.5, 1 and 2 mg), Kalma® (0.5, 1 and 2 mg), Xanax® (1 and 2 mg), Mylan® 2 mg and Alprazolam Sandoz 2 mg. The DSG do not make a determination of legitimacy when tablets expected to contain alprazolam are found to contain alprazolam, as the tablets may still be counterfeit as opposed to a diverted pharmaceutical product. Variables extracted from the DSG database included month of the offence, product description, analytical detections and the number of detections.

### Analytical methodology

Drug exhibits submitted to the DSG are visually examined and tablets expected to contain alprazolam are photographed. Representative sampling methods are employed to ensure the reliable reporting of results. Drug exhibits are analysed by gas chromatography–mass spectrometry (GC–MS) and certified reference materials (CRMs) are required for confirmation. Where a CRM for a particular drug is unavailable, a reputable literature reference is utilised to indicate the presence of a drug. The DSG database is updated when newly detected drugs are encountered. During the study period there were 31 benzodiazepines (pharmaceutical or NPS) listed on the DSG database (Table S1).

### Clinical data

The Emerging Drugs Network of Australia – Victoria (EDNAV) project is a multi‐institutional, prospective clinical study involving the comprehensive analysis of blood samples obtained from a purposive sample of individuals presenting to Victorian emergency departments with illicit drug‐related toxicity [14]. Under an ethics committee approved waiver of consent (HREC/66506/Austin‐2020), de‐identified clinical and analytical data are collated within a secure online Research Electronic Data Capture (REDCap) database (EDNAV Clinical Registry) [15, 16]. The full methodology has been published in detail previously [14]. During the study period, the EDNAV project was operational across 16 public hospital emergency departments in Victoria (13 metropolitan and three regional emergency departments). Additionally, the age of eligibility was reduced to include patients aged 12 years and above in May 2022 for select EDNAV hospital sites.

### Analytical methodology

Clinical blood samples from eligible cases were sent to the Victorian Institute of Forensic Medicine Toxicology Department for comprehensive toxicological analysis. Blood samples were analysed via two separate liquid chromatography–tandem mass spectrometric (LC–MS/MS) screens, totalling 575 pharmaceutical, illicit and novel substances [17]. All blood samples subsequently underwent untargeted screening via liquid chromatography–quadrupole time‐of‐flight mass spectrometry (LC Q‐TOF MS) using a combination of in‐house targets and the crowd‐sourced HighResNPS.com database [18, 19]. At the time of analysis, a total of 38 benzodiazepines and benzodiazepine metabolites were being monitored (Table S1). Clobromazolam (phenazolam) was formally added to the screen in May 2022, with cases prior to April 2022 not screened for the presence of clobromazolam.

### Case selection and extracted variables

The EDNAV Clinical Registry was systematically searched for reported alprazolam exposure, either on emergency department presentation or upon patient recovery between project commencement (September 2020) and August 2022. A total of 128 cases reported exposure to an alprazolam‐containing product on hospital admission or upon recovery. Three cases were known to be regularly prescribed alprazolam and therefore were excluded as the ingested alprazolam was likely sourced legally. Variables extracted from the EDNAV Clinical Registry for this study included demographics (age, sex), reported drug use and circumstances (location, intent), and analytical detections.

### Clinical and toxicological data interpretation

Toxicological results for all cases were reviewed for medications administered as part of medical management (pre‐hospital and in hospital), reported drug exposures and regularly prescribed medications, to exclude erroneous conclusions of an acute drug exposure. Any benzodiazepine that aligned with a regularly prescribed medication or was specifically reported as an exposure proximate to hospital presentation (separate to the reported use of an alprazolam‐containing product) was excluded from subsequent analysis. Finally, all benzodiazepine detections were reviewed to confirm that their presence was a true detection rather than associated with the metabolism of another benzodiazepine. The detection of desalkylflurazepam was excluded if reported in the setting of midazolam administration [20]. Similarly, a benzodiazepine‐type NPS was excluded if detected in the presence of a known parent drug [21]. Illicit drugs co‐detected with benzodiazepine‐type NPSs were classified into pharmacological effect groups as defined by the United Nations Office on Drugs and Crime [22].

### Data analysis

Clinical and analytical data were extracted and analysed using Microsoft Excel® 16.65, (Microsoft, Redmond, WA, USA) and GraphPad Prism® 9.4.1 (GraphPad Software, LLC, San Diego, CA, USA). Descriptive statistics were generated for analytical and demographic variables. Changes in drug detections over time were analysed visually based on the month of detection for seizure data or hospital presentation. Patterns of co‐detected non‐prescribed drug detections were analysed based on substance effect group classification. The full list of detected substances for seized tablets and clinical cases is detailed in Table S2.

## RESULTS

### Police data for suspect counterfeit alprazolam tablets

Victoria Police analysed 623 individual seizures of suspect counterfeit alprazolam tablets, ranging from single tablets to the largest individual seizure involving 8893 tablets. This accounted for a total of 35 068 individual tablets. Seized products were commonly labelled with pharmaceutical brand names including ‘Xanax®’ (266 seizures, 20 920 tablets), ‘Kalma®’ (196 seizures, 9943 tablets), ‘Mylan®’ (124 seizures, 3906 tablets) and ‘Alprax®’ (34 seizures, 293 tablets). The suspect seized alprazolam tablets were predominately labelled with a 2 mg tablet strength (94.9%).

**FIGURE 1:**
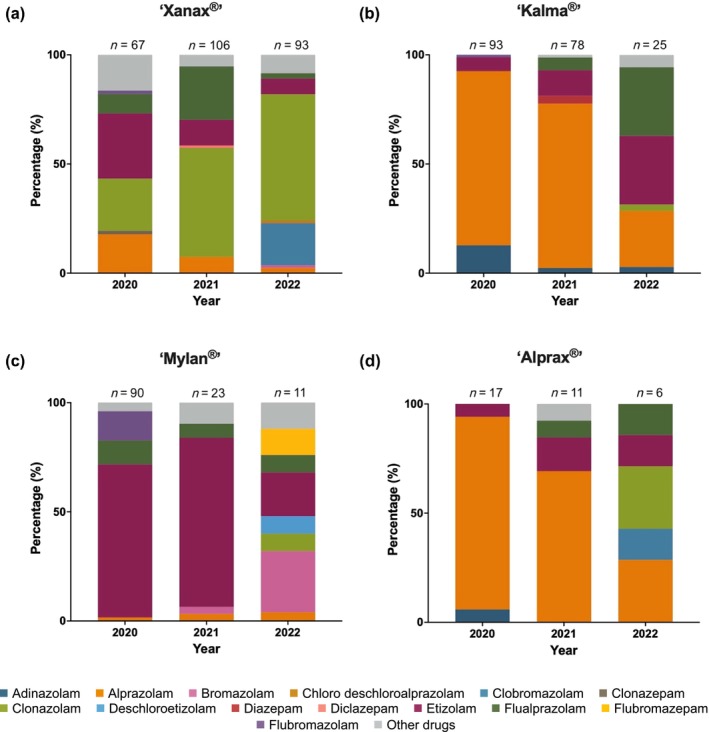
Composition of suspect counterfeit alprazolam tablets analysed by Victoria Police between March 2020 and August 2022: (a) ‘Xanax®’ (266 seizures, 20 920 tablets); (b) ‘Kalma®’ (196 seizures, 9943 tablets); (c) ‘Mylan®’ (124 seizures, 3906 tablets); and (d) ‘Alprax®’ (34 seizures, 293 tablets). Other drugs include: amantadine, atropine, benzocaine, cyproheptadine, desmethyltramadol, diphenhydramine, doxepin, etodesnitazene, ketamine, lidocaine, mestanolone, *N*‐ethylyheptedrone, oxycodone, oxymetholone, promethazine and quetiapine.

Tablets containing alprazolam in the absence of any other substance were identified in 30.3% of all seizures (*n* = 189/623). The remaining seizures contained a drug(s) in addition to alprazolam (*n* = 11), a different benzodiazepine(s) (*n* = 343), a different benzodiazepine(s) and another drug(s) (*n* = 26) or did not contain a benzodiazepine (*n* = 8). No active drug was detected in a total of 46 seizures (7.4%). A benzodiazepine‐type NPS was detected in 375 seizures. The presence of an alprazolam‐only containing tablet accounted for 38.0% (*n* = 101/266) and 33.8% (n = 75/222) of seizures in 2020 and 2021, respectively, but dropped in the first 8 months of 2022 to 9.6% (*n* = 13/135). The tablet composition varied between brands (Figure 1). The lowest rates of tablet adulteration/substitution were present in ‘Kalma®’ (26.0%) and ‘Alprax®’ (29.4%) tablets (Figure 1).

### Clinical cases of non‐fatal alprazolam intoxication

Exposure to non‐prescribed alprazolam was reported in 11.2% (*n* = 125/1112) of all EDNAV cases over the study period. The majority of cases identified as male (*n* = 86, 68.8%), with a median age of 26 years (IQR 18–31 years, range 16–68 years) (Table 1). The documented use intent for alprazolam was most commonly recreational (*n* = 68, 54.4%) and occurred within a private residence (*n* = 68, 54.4%) (Table 1). A prescription medication history was known in 47.2% (*n* = 59) of cases, of which 11 were regularly prescribed a benzodiazepine other than alprazolam.

**TABLE 1:** Demographics of cases reporting alprazolam use from the Emerging Drugs Network of Australia – Victoria (EDNAV) project, September 2020–August 2022 (*n* = 125).

Median age (IQR, range), years	26 (18–31, 16–68)
**Sex, *n* (%)**	
Male	86 (68.8)
Female	39 (31.2)
**Exposure setting, *n* (%)**	
Private residence	67 (53.6)
Public environment	36 (28.8)
Unknown	10 (8.0)
Other^a^	8 (6.4)
Private event/party	3 (2.4)
Licenced venue	1 (0.8)
**Alprazolam‐containing product reported, *n* (%)**	
‘Xanax®’	87 (69.6)
‘Alprazolam’/‘street alprazolam’	35 (28)
‘Mylan®’	3 (2.4)
**Drug use intent, *n* (%)**	
Recreational	68 (54.4)
Deliberate self‐poisoning	23 (18.4)
Unknown	31 (24.8)
Other^b^	3 (2.4)
**Co‐detected substances** ^c^	
Stimulant(s)	80 (64.0)
Opioid(s)	41 (32.8)
Cannabinoid(s)	30 (24.0)
Depressant(s)	15 (14.7)
Dissociative(s)	7 (5.6)
Other novel psychoactive substance(s)	3 (2.4)
Ethanol^d^	11 (14.7)

^a^ Other exposure, including correctional facility, pharmacy, supported residential services and hotel.

^b^ Other intent, includes suspected drink spiking and analgesia.

^c^ Drug classification: stimulant(s) – cocaine, methylamphetamine, 3,4‐methylenedioxymethamphetamine; depressant(s) – gamma‐hydroxybutrate (not tested in 23 cases, *n* = 102); dissociative(s) – ketamine; cannabinoid(s) – delta‐9‐tetrahydrocannabinol; opioid(s) – 6‐monoacetylmorphine (heroin metabolite), buprenorphine, codeine, morphine, fentanyl, methadone, oxycodone, tramadol; other new psychoactive substances – methylone, 4‐chloromethcathinone, 5F‐CUMYL‐PINACA.

^d^ Ethanol – not measured in 50 cases (*n* = 75).

‘Xanax®’ was the most frequently referenced preparation of alprazolam reportedly used (*n* = 87) (Table 1). Of the 37 presentations where a drug description was documented, 36 reported the use of a tablet and the remaining case reported a wafer. The strength of alprazolam ingested was seldom documented. Seven cases reported the use of a 2 mg tablet, and one case reported the use of a 1 mg tablet.

Alprazolam was detected in 19 cases. One or more benzodiazepine‐type NPSs were detected in 78.4% (*n* = 98) of cases. Excluding benzodiazepine detections where it was a known regular medication or additionally reported as an acute exposure, there were a total of nine cases where no additional benzodiazepine was detected. There was no consistent pattern of drug detection across these cases. At least one other illicit drug (median = 1, range = 1–5) was co‐detected in 74.4% (*n* = 93) of the cases, including a psychostimulant (methylamphetamine, *n* = 70; cocaine, *n* = 17; or 3,4‐methylenedioxymethamphetamine, *n* = 10), cannabis (*n* = 30) or heroin (*n* = 11) (Table 1).

### Comparison of police and clinical data

**FIGURE 2:**
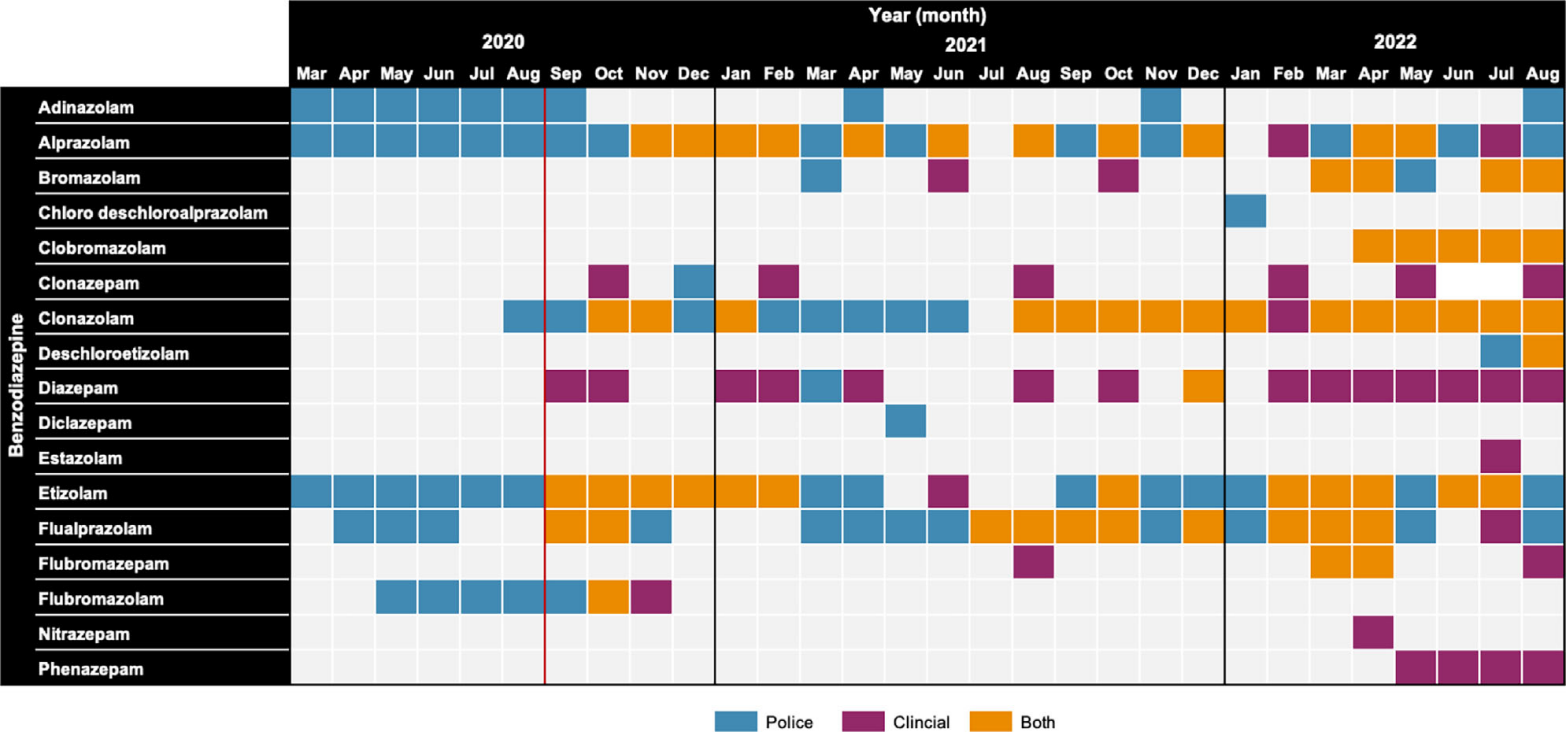
Detections of prescription benzodiazepines and benzodiazepine‐type new psychoactive substances (NPSs) in police data (March 2020–August 2022, *n* = 569 seizures) and clinical data from the Emerging Drugs Network of Australia – Victoria (EDNAV) project (September 2020–August 2022, *n* = 116 cases). The red vertical line represents the commencement of EDNAV project sampling. Blue represents detections in police data alone, purple represents detection in EDNAV clinical data alone, and orange represents detection in both police and EDNAV clinical data. Prescription benzodiazepine detections for EDNAV data exclude where the benzodiazepine was a known regular medication, reported as an acute exposure or reported as part of the acute hospital presentation. Clobromazolam was formally added to the Victorian Institute of Forensic Medicine analytical toxicology screen in May 2022; only EDNAV cases from April 2022 onwards have been analysed for the presence of clobromazolam.

In the 6‐month period prior to the commencement of the EDNAV project (March–August 2020), suspect counterfeit alprazolam seizures predominately consisted of etizolam (*n* = 89 seizures) and alprazolam (*n* = 72 seizures) (Figure 2).

In the period from September to December 2020, alprazolam (*n* = 32/88, 36.4%) and etizolam (*n* = 27/88, 30.7%) were the most commonly detected benzodiazepines in the police data. Over the same 4‐month period in the clinical data, etizolam was the most frequently detected benzodiazepine (*n* = 11/19, 57.9%). Clonazolam emerged in the last quarter of 2020 in both data sets (Figure 2). Moving into 2021, alprazolam remained the most frequently detected benzodiazepine in the police data; however, detections of clonazolam (*n* = 47/222 seizures, 21.2%) and etizolam (*n* = 47/222 seizures, 21.2%) increased. Clinical data similarly saw a shift to clonazolam (*n* = 10/26 cases, 38.7%) in 2021. Clonazolam was the most frequently detected benzodiazepine in 2022 in police data (*n* = 53/135 seizures, 39.3%) and EDNAV data (*n* = 38/169 cases, 22.5%). The emergence of clobromazolam occurred concurrently in both data sets in April 2022 and continued to be detected throughout the remainder of the 2022 study period (Figure 2). The majority of benzodiazepines were concurrently detected across both data sets, with the exception of adinazolam, chloro deschloroalprazolam and diclazepam, which were only identified in police data, and estazolam, nitrazepam and phenazepam, which were only identified in EDNAV data.

### Benzodiazepine‐type NPS combinations

**FIGURE 3:**
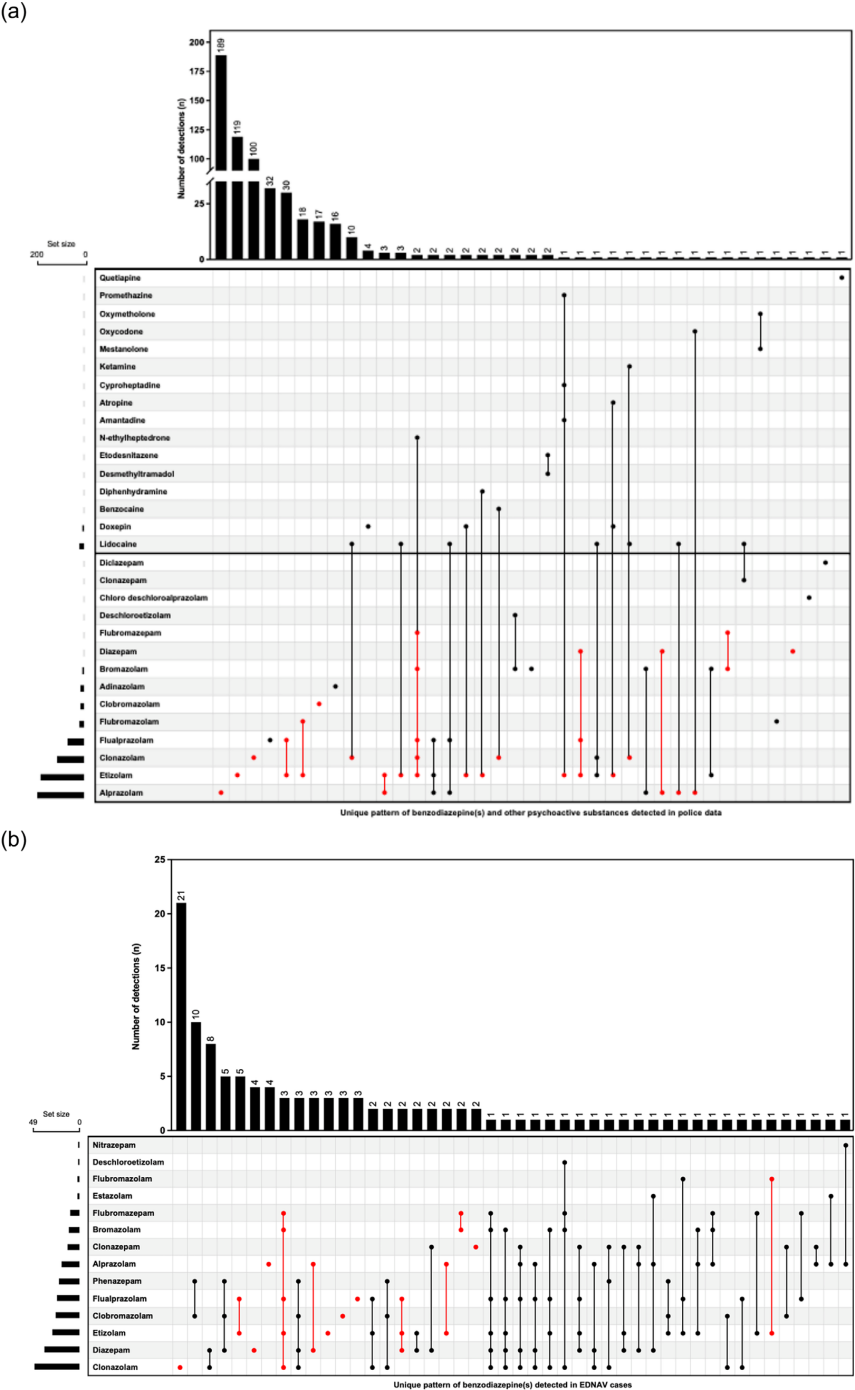
Unique combinations of drugs detected over the period September 2020–August 2022 with: (a) police data, including non‐benzodiazepine drugs (*n* = 39 combinations); and (b) Emerging Drugs Network of Australia – Victoria (EDNAV) clinical data (*n* = 46 combinations). The red coloured sections highlight where a detection pattern of a benzodiazepine(s) was consistent between police and clinical data.

Within the police data there were a total of 39 different combinations of drugs, including non‐benzodiazepine substances, detected over the 2‐year period (Figure 3a). Eighty‐one percent of tablets only contained a single benzodiazepine. A median of two drugs (range = 1–6) were detected per tablet, with a median of one benzodiazepine per tablet (range = 0–5). Two or more benzodiazepines were concurrently detected in 10.6% of seizures. The most frequently detected tablet compositions were single drug detections: alprazolam (*n* = 189 seizures, 30.3%), etizolam (*n* = 119 seizures, 19.1%), clonazolam (*n* = 100 seizures, 16.1%), flualprazolam (*n* = 32 seizures, 5.1%) and etizolam plus flualprazolam (*n* = 30 seizures, 4.8%).

There were a total of 46 different combinations of unreported benzodiazepines detected within EDNAV cases (Figure 3b). There was a median of two unreported benzodiazepines detected per case (range = 1–6). As opposed to the police data where 80.7% of tablets contained a single benzodiazepine, the detection of a single unreported benzodiazepine was only evident in 39.2% of EDNAV cases. The most frequent detections included clonazolam (*n* = 21 cases, 16.8%), clobromazolam plus phenazepam (*n* = 10 cases, 8.0%), clonazolam plus diazepam (*n* = 8 cases, 6.4%), etizolam plus flualprazolam (*n* = 5 cases, 4.0%) and clobromazolam plus phenazepam plus diazepam (*n* = 5 cases, 4.0%).

Fourteen patterns of benzodiazepine co‐detections were common between the police and EDNAV data sets (Figure 3). The largest combination of concomitantly detected benzodiazepines was the combination of bromazolam, clonazolam, etizolam, flualprazolam and flubromazepam. This combination was only observed in two seized tablets, one each in March and April of 2022, in combination with the synthetic cathinone *N*‐ethylheptedrone. This combination of the same five benzodiazepine‐type NPSs was detected in four EDNAV cases in April 2022, including one where diazepam was additionally detected. However, neither *N*‐ethylheptedrone nor any other cathinone was detected in any of the four EDNAV cases.

### Presence of substituents and adulterants

Police data were reviewed to identify non‐benzodiazepine substances present within seized samples, either in combination with or in place of a benzodiazepine. The most frequently co‐detected substance was lidocaine (*n* = 19 seizures, 808 tablets). The remaining adulterants were only detected in a small number of seizures (Table S2). There were eight seizures (55 tablets) where a benzodiazepine was not detected. This included the detection of etodesnitazene and desmethyltramadol in two seizures.

## DISCUSSION

Counterfeit alprazolam appears to be a relatively new trend in the Australian illicit drug market, with few reports of benzodiazepine‐type NPSs preceding 2019 [23, 24, 25, 26]. We sought to better contextualise the formulation and use of suspect counterfeit benzodiazepine products in the Australian community. The current work demonstrated the highly variable composition of alprazolam products that were detected in both police and clinical data. The detection of benzodiazepines and/or other psychoactive substances appeared to fluctuate over time, with a similar detection profile identified in police and clinical data. This has important public health implications, as non‐medical benzodiazepine use and the emergence of benzodiazepine‐type NPSs have both been identified as global public health threats [27, 28, 29].

Alprazolam was only identified in a third of all seized suspect counterfeit alprazolam products and less than a fifth of hospital presentations with a reported acute ingestion of an alprazolam‐containing product. Although a variety of branded alprazolam products were identified in seizures, ‘Xanax®’ was reported in the majority (69.6%) of EDNAV cases. It is unclear as to the extent of consumer awareness of the contents of non‐prescription benzodiazepines in an Australian context. It is possible that, although no longer a brand marketed in Australia, Xanax® has recognisability within the Australian population through frequent references in popular culture [30]. In surveys of sentinel populations of people who use drugs, only 1%–2% of respondents reported recent benzodiazepine‐type NPS use [31, 32]. Rather, these individuals most commonly reported accessing branded or generic diazepam or alprazolam [12].

The police and clinical data highlight the large variability in tablet composition within suspect counterfeit alprazolam tablets. This was most evident with the shift in detections from etizolam to clonazolam, followed by clobromazolam (Figure 3). This pattern was similarly identified in drug seizure, clinical and coronial data from other Australian jurisdictions in a similar time frame [2, 23, 24]. Benzodiazepine‐type NPS products appear to be largely imported from laboratories in China [21, 33], and so a degree of congruency with international markets is not unexpected. This pattern might be related to the World Health Organization recommending the scheduling of etizolam in December 2019 [34]. Clonazolam was subsequently recommended for scheduling in December 2020 [35]. There have been few reports of clobromazolam outside of Australia following the initial detection in a seizure from Sweden in March 2016 [36, 37]. Clobromazolam was reviewed for scheduling by the UK but was not recommended owing to the low prevalence in the community [36].

Most of the analysed tablets only contained a single benzodiazepine (80.7%), which was consistent with seizure data from Queensland and New South Wales (NSW) [2, 24]. This contrasted with EDNAV data, where 60.8% of presentations involved the co‐detection of two or more benzodiazepines and 44.0% involved the detection of two or more benzodiazepine‐type NPSs. The co‐detection of benzodiazepine‐type NPSs has also been reported in 27.5% of coronial cases in Australia and 11.5% of the Swedish ‘STRIDA’ cohort [23, 38]. The sampling framework, geography and time frame makes direct comparison difficult, but may be an indication of individuals concurrently using multiple benzodiazepine products from various sources. For instance, diazepam was detected in 30.4% of EDNAV cases but only from three seizures in the police data. Diazepam is the most frequently prescribed subsidised benzodiazepine in Australia [39] and is also more likely to be sourced via a diverted prescription, compared with alprazolam [12]. Conversely, some of these co‐detections could reflect an uncharacterised metabolic pathway of a benzodiazepine‐type NPS giving rise to benzodiazepine intermediate metabolites.

A non‐benzodiazepine drug(s) was present within 7.2% of suspect counterfeit alprazolam tablets analysed, none of which are known excipients of pharmaceutical‐grade alprazolam. The most frequently detected drug was lidocaine, which is a known bulking agent in illicit drug manufacture [2, 40]. There were three seizures involving the detection of an opioid (oxycodone, etodesnitazene and desmethyltramadol). Opioid adulterated/substituted products have been described previously in North America, including counterfeit ‘Xanax®’ tablets, leading to life‐threatening opioid toxicity and fatality in one instance [6, 41]. Analysis of benzodiazepine seizure data from Queensland and NSW did not identify opioid adulteration during the study period. However, the NSW government subsequently released a drug alert about counterfeit ‘Kalma®’ tablets that contained etodesnitazene and *O*‐desmethyltramadol in August 2022 [42]. It is difficult to predict the clinical impact of ingestion without knowledge of the concentrations within the tablets. From a public health perspective, opioid adulteration of benzodiazepines is concerning because of the risk of unexpected opioid toxicity in a population that might not have ready access to or familiarity with naloxone.

The presence and unpredictability of non‐prescription benzodiazepine products is an ongoing public health challenge owing to the unregulated nature of their use. In an acute setting, patients could inadvertently experience toxicity from use depending on the potency and half‐life of the benzodiazepine(s) contained within a tablet [2]. Currently it appears that benzodiazepine‐type NPSs are having a modest impact in coronial cases in Victoria, with a benzodiazepine‐type NPS detected in 7.3% of overdose fatalities, compared with the 39.5% that contained diazepam [43]. Rather, prescription benzodiazepines as a class continue to be detected in approximately half of all drug overdose fatalities annually over the past decade [26, 44]. However, there are some concerns that additional regulatory controls may impact the ability for individuals to access prescription medications like benzodiazepines, making unregulated benzodiazepines a feasible, but unintended alternative [12, 45].

There are several limitations related to this research. Police data were based on drug items expected to contain alprazolam based on visual appearance and, therefore, no determination on legitimacy was provided. Additionally, the EDNAV project employs purposive sampling, therefore only representing a subset of patients presenting to hospital with illicit drug‐related toxicity. A prescription medication history was not obtained for all cases, nor was it known if cases were obtaining additional benzodiazepines from other sources (i.e. diverted medications). Therefore, we were not able to delineate these detections from those that were associated with the reported alprazolam product with complete certainty. Owing to the de‐identification process, repeat hospital attendances could not be determined over the study period. Analysis of the police and clinical data utilised similar analytical methodology but occurred in two separate laboratories. Detections in the seizure data and the patient blood samples were not quantified, and therefore it is difficult to determine the contribution of any one benzodiazepine to the reported toxicity. These caveats should be considered when reviewing the presented data, with the possibility that a broader range of benzodiazepine‐type NPSs are in circulation within the community.

## CONCLUSION

Our findings highlight the range of NPSs and/or prescription benzodiazepines that may be present within suspect counterfeit alprazolam products in Australia. Police data demonstrate complementarity with detections from clinical cases, highlighting a fluctuating pattern of detections over time. Concerningly, individuals appear to be unaware of the composition of unregulated benzodiazepine products. Continued toxicosurveillance efforts utilising complementary data sets are imperative to provide timely drug intelligence to key community stakeholders and identify evidence of emergent drug trends to facilitate early intervention.

## AUTHOR CONTRIBUTIONS

**Rebekka Syrjanen:** Data curation (equal); formal analysis (lead); project administration (equal); writing – original draft (equal); writing – review and editing (equal). **Shaun L. Greene:** Conceptualization (lead); funding acquisition (lead); methodology (lead); project administration (equal); supervision (supporting); writing – original draft (equal); writing – review and editing (equal). **Sarah E. Hodgson:** Investigation (equal); writing – review and editing (equal). **Rachelle Abouchedid:** Investigation (equal); writing – review and editing (equal). **Dimitri Gerostamoulos:** Investigation (equal); supervision (supporting); writing – review and editing (equal). **Christie Magee:** Data curation (equal); formal analysis (supporting); writing – review and editing (equal). **Melissa Bremner:** Supervision (equal); validation (equal); writing – review and editing (equal). **Jennifer L. Schumann:** Investigation (supporting); supervision (lead); writing – original draft (equal); writing – review and editing (equal).

## DECLARATION OF INTERESTS

None declared.

## Supporting information


**Table S1.** List of monitored benzodiazepines and benzodiazepine metabolites at Victoria Police and the Victorian Institute of Forensic Medicine.
**Table S2.** Summary of the analytical detections in Victoria Police seizure data by number of cases and by number of tablets (March 2020–August 2022, *n* = 623 cases, *n* = 35 068 tablets) and in clinical data from the Emerging Drugs Network of Australia – Victoria (EDNAV) project (September 2020–August 2022, *n* = 125 cases).

## Data Availability

The data that support the findings of this study may be available on request from the corresponding author. The data are not publicly available due to privacy or ethical restrictions.

## References

[1] Organisation for Economic Co‐operation and Development European Union Intellectual Property Office . Trade in Counterfeit Pharmaceutical Products [Internet]. 2020. Accessed Aug 04, 2024. https://www.oecd-ilibrary.org/content/publication/a7c7e054-en

[2] Blakey K , Thompson A , Matheson A , Griffiths A . What's in fake 'Xanax'?: A dosage survey of designer benzodiazepines in counterfeit pharmaceutical tablets. Drug Test Anal. 2022;14(3):525–530. 10.1002/dta.3119 34170084

[3] Therapeutic Goods Administration . Counterfeit Alprazolam 2mg and Kalma 2 tablets [Internet] Canberra (AU): Commonwealth of Australia Department of Health; 2020 [Accessed Aug 04, 2024. Available from: https://www.tga.gov.au/alert/counterfeit-alprazolam-2mg-and-kalma-2-tablets

[4] Tracy DK , Wood DM , Baumeister D . Novel psychoactive substances: types, mechanisms of action, and effects. BMJ. 2017;356:i6848. 10.1136/bmj.i6848 28122697

[5] Tracy DK , Wood DM , Baumeister D . Novel psychoactive substances: identifying and managing acute and chronic harmful use. BMJ. 2017;356:i6814. 10.1136/bmj.i6814 28122703

[6] Arens AM , van Wijk XM , Vo KT , Lynch KL , Wu AH , Smollin CG . Adverse Effects From Counterfeit Alprazolam Tablets. JAMA Intern Med. 2016;176(10):1554–1555. 10.1001/jamainternmed.2016.4306 27532131

[7] Bollinger K , Weimer B , Heller D , Bynum N , Grabenauer M , Pressley D , et al. Benzodiazepines reported in NFLIS‐Drug, 2015 to 2018. Forensic Sci Int Synerg. 2021;3:100138. 10.1016/j.fsisyn.2021.100138 33665593 PMC7905184

[8] Victorian Government Department of Health . High potency benzodiazepine tablets (May 2022) [Internet] Melbourne (AU): Victorian Government Department of Health; 2022 [Accessed May 25, 2024. Available from: https://www.health.vic.gov.au/drug-alerts/high-potency-benzodiazepine-tablets

[9] Therapeutic Goods Administration . Cancellations by sponsors: XANAX alprazolam 1mg tablet blister pack Cancelled under Section 30(1)(c) of the Act [Internet] Canberra (AU): Therapeutic Goods Administration; 2023 [Accessed Sep 12, 2024. Available from: https://www.tga.gov.au/resources/cancellations-by-sponsors/xanax-alprazolam-1mg-tablet-blister-pack-cancelled-under-section-301c-act

[10] Essink S , Nugteren‐van Lonkhuyzen JJ , van Riel A , Dekker D , Hondebrink L . Significant toxicity following an increase in poisonings with designer benzodiazepines in the Netherlands between 2010 and 2020. Drug Alcohol Depend. 2022;231:109244. 10.1016/j.drugalcdep.2021.109244 34998250

[11] Carpenter JE , Murray BP , Dunkley C , Kazzi ZN , Gittinger MH . Designer benzodiazepines: a report of exposures recorded in the National Poison Data System, 2014‐2017. Clin Toxicol (Phila). 2019;57(4):282–286. 10.1080/15563650.2018.1510502 30430874

[12] Grigg J , Peacock A , Lenton S , Salom C , Agramunt S , Thomas N , et al. Real or fake? Sourcing and marketing of non‐prescribed benzodiazepines amongst two samples of people who regularly use illicit drugs in Australia. Drug Alcohol Rev. 2023;42(6):1559–1565. 10.1111/dar.13722 37490407 PMC10947514

[13] Syrjanen R , Greene SL , Weber C , Smith JL , Hodgson SE , Abouchedid R , et al. Characteristics and time course of benzodiazepine‐type new psychoactive substance detections in Australia: results from the Emerging Drugs Network of Australia ‐ Victoria project 2020‐2022. Int J Drug Policy. 2023;122:104245. 10.1016/j.drugpo.2023.104245 37944339

[14] Syrjanen R , Schumann J , Fitzgerald J , Gerostamoulos D , Abouchedid R , Rotella JA , et al. The Emerging Drugs Network of Australia ‐ Victoria Clinical Registry: A state‐wide illicit substance surveillance and alert network. Emerg Med Australas. 2022;35(1):82–88. 10.1111/1742-6723.14059 36053993

[15] Harris PA , Taylor R , Minor BL , Elliott V , Fernandez M , O'Neal L , et al. The REDCap consortium: Building an international community of software platform partners. J Biomed Inform. 2019;95:103208. 10.1016/j.jbi.2019.103208 31078660 PMC7254481

[16] Harris PA , Taylor R , Thielke R , Payne J , Gonzalez N , Conde JG . Research electronic data capture (REDCap)‐‐a metadata‐driven methodology and workflow process for providing translational research informatics support. J Biomed Inform. 2009;42(2):377–381. 10.1016/j.jbi.2008.08.010 18929686 PMC2700030

[17] Di Rago M , Pantatan S , Hargreaves M , Wong K , Mantinieks D , Kotsos A , et al. High Throughput Detection of 327 Drugs in Blood by LC‐MS‐MS with Automated Data Processing. J Anal Toxicol. 2021;45(2):154–183. 10.1093/jat/bkaa057 32451548

[18] von Cüpper M , Dalsgaard PW , Linnet K . Identification of New Psychoactive Substances in Seized material Using UHPLC‐QTOF‐MS and An Online Mass Spectral Database. J Anal Toxicol. 2021;44(9):1047–1051. 10.1093/jat/bkaa028 32232329

[19] Mardal M , Andreasen MF , Mollerup CB , Stockham P , Telving R , Thomaidis NS , et al. HighResNPS.com: An Online Crowd‐Sourced HR‐MS Database for Suspect and Non‐targeted Screening of New Psychoactive Substances. J Anal Toxicol. 2019;43(7):520–527. 10.1093/jat/bkz030 31095696

[20] Vogt S , Kempf J , Buttler J , Auwärter V , Weinmann W . Desalkylflurazepam found in patients' samples after high‐dose midazolam treatment. Drug Test Anal. 2013;5(9–10):745–747. 10.1002/dta.1484 23713025

[21] European Monitoring Centre for Drugs and Drug Addiction . New benzodiazepines in Europe – a review [Internet] Luxembourg (LU): Publications Office of the European Union; 2021. Accessed Oct 12, 2024. p. 48. https://www.emcdda.europa.eu/publications/rapid-communications/new-benzodiazepines-europe-review_en

[22] United Nations Office on Drugs and Crime . UNODC Early Warning Advisory on New Psychoactive Substances: Pharmacology [Internet] Vienna (AT): United Nations Office on Drugs and Crime; 2022 [Accessed Nov 23, 2024. Available from: https://www.unodc.org/LSS/Page/NPS/pharmacology

[23] Darke S , Peacock A , Duflou J , Farrell M , Lappin J . Characteristics of fatal 'novel' benzodiazepine toxicity in Australia. Forensic Sci Int. 2022;331:111140. 10.1016/j.forsciint.2021.111140 34894611

[24] Brown J . Unregistered benzodiazepines and counterfeit alprazolam: trends in NSW Sydney (AU): National Drug & Alcohol Research Centre; 2022.

[25] Bade R , Stockham P , Painter B , Celma A , Bijlsma L , Hernandez F , et al. Investigating the appearance of new psychoactive substances in South Australia using wastewater and forensic data. Drug Test Anal. 2019;11(2):250–256. 10.1002/dta.2484 30129282

[26] Coroners Court of Victoria . Victorian overdose deaths, 2011–2020 [Internet] Melbourne (AU): Coroners Court of Victoria; 2021. Accessed Apr 01, 2023. p. 25.

[27] European Monitoring Centre for Drugs and Drug Addiction . European Drug Report 2023: Trends and Developments [Internet] Lisbon (PT): European Monitoring Centre for Drugs and Drug Addiction; 2023. Accessed Oct 23, 2023. https://www.emcdda.europa.eu/publications/european-drug-report/2023_en

[28] United Nations Office on Drugs and Crime . Non‐medical use of benzodiazepines: a growing threat to public health? [Internet] Vienna (AT): United Nations Office on Drugs and Crime; 2017. Accessed Oct 13, 2024. p. 12.

[29] Health AIo, Welfare . Alcohol, tobacco & other drugs in Australia [Internet] Canberra (AU): AIHW; 2023 Accessed. https://www.aihw.gov.au/reports/alcohol/alcohol-tobacco-other-drugs-australia

[30] Stickle B . A High Note: Drug Misuse in Popular Rap Music. Subst Use Misuse. 2021;56(10):1448–1456. 10.1080/10826084.2021.1936046 34121603

[31] Sutherland R , Karlsson A , King C , Jones F , Uporova J , Price O , et al. Australian Drug Trends 2022: Key Findings from the National Ecstasy and Related Drugs Reporting System (EDRS) Interviews [Internet] Sydney (AU): National Drug and Alcohol Research Centre, UNSW Sydney; 2022. p. 106.

[32] Sutherland R , Uporova J , King C , Jones F , Karlsson A , Gibbs D , et al. Australian Drug Trends 2022: Key Findings from the National Illicit Drug Reporting System (IDRS) Interviews. [Internet] Sydney (AU): National Drug and Alcohol Research Centre, University of New South Wales: National Drug and Alcohol Research Centre, UNSW Sydney; 2022 Accessed.

[33] Australian Criminal Intelligence Commission . Illicit Drug Data Report 2019–20 [Internet] Canberra (AU): Australian Criminal Intelligence Commission; 2021. Accessed Dec 22, 2023. p. 220. https://www.acic.gov.au/publications/illicit-drug-data-report/illicit-drug-data-report-2019-20

[34] World Health Organization . WHO Expert Committee on Drug Dependence: forty‐second report [Internet] Geneva (CH); 2020. Accessed Jan 10, 2024. p. 44. https://www.who.int/publications/i/item/9789240001848

[35] World Health Organization . WHO Expert Committee on Drug Dependence: forty‐third report [Internet] Geneva (CH): World Health Organization; 2021. Accessed Jan 10, 2024. p. 205. https://www.who.int/publications/i/item/9789240023024

[36] Advisory Council on the Misuse of Drugs . Novel Benzodiazepines: A review of the evidence of use and harms of Novel Benzodiazepines [Internet] London (GB); 2020. Accessed Dec 02, 2023. p. 44. https://www.gov.uk/government/publications/novel-benzodiazepines-prevalence-and-harms-in-the-uk

[37] Castle JW , Syrjanen R , Di Rago M , Schumann JL , Greene SL , Glowacki LL , et al. Identification of clobromazolam in Australian emergency department intoxications using data‐independent high‐resolution mass spectrometry and the HighResNPS.com database. J Anal Toxicol. 2024;48(5):273–280. 10.1093/jat/bkae012 38459915

[38] Bäckberg M , Pettersson Bergstrand M , Beck O , Helander A . Occurrence and time course of NPS benzodiazepines in Sweden ‐ results from intoxication cases in the STRIDA project. Clin Toxicol (Phila). 2019;57(3):203–212. 10.1080/15563650.2018.1506130 30348014

[39] Australian Institute of Health and Welfare . Alcohol, tobacco & other drugs in Australia [Internet] Canberra: AIHW; 2022. Accessed Oct 04, 2024. https://www.aihw.gov.au/reports/alcohol/alcohol-tobacco-other-drugs-australia

[40] Peck Y , Clough AR , Culshaw PN , Liddell MJ . Multi‐drug cocktails: Impurities in commonly used illicit drugs seized by police in Queensland, Australia. Drug Alcohol Depend. 2019;201:49–57. 10.1016/j.drugalcdep.2019.03.019 31181437

[41] Chapman BP , Lai JT , Krotulski AJ , Fogarty MF , Griswold MK , Logan BK , et al. A Case of Unintentional Opioid (U‐47700) Overdose in a Young Adult After Counterfeit Xanax Use. Pediatr Emerg Care. 2021;37(9):e579–e580. 10.1097/pec.0000000000001775 30789871

[42] New South Wales Ministry of Health . Fake Kalma alprazolam tablets found to contain strong opioids [Internet] Sydney (AU): New South Wales Ministry of Health; 2022 [Accessed 24/10/2022. Available from: https://www.health.nsw.gov.au/aod/public-drug-alerts/Pages/fake-kalma-contains-strong-opioids-aug2022.aspx

[43] Coroners Court of Victoria . Victorian overdose deaths, 2013–2022 [Internet] Melbourne (AU): Coroners Court of Victoria; 2023. Accessed Nov 11, 2024. p. 24. https://coronerscourt.vic.gov.au/victorian-overdose-deaths-2013-2022

[44] Lloyd B , Dwyer J , Bugeja L , Jamieson A . Alprazolam in fatal overdose following regulatory rescheduling: A response to Deacon et al. Int J Drug Policy. 2017;39:138–139. 10.1016/j.drugpo.2016.10.008 27856134

[45] McAuley A , Matheson C , Robertson JR . From the clinic to the street: the changing role of benzodiazepines in the Scottish overdose epidemic. Int J Drug Policy. 2022;100:103512. 10.1016/j.drugpo.2021.103512 34753047

