# Supplementary material for: Counterfeit ‘Xanax®’ tablets: A comparative study of clinical and seizure data in Victoria, Australia
Source: Addiction. 2025 Aug 26;120(12):2465–75. doi: 10.1111/add.70174 (PMC12586763; doi:10.1111/add.70174)
Supplement: Supplementary file 1 — Table S1. List of monitored benzodiazepines and benzodiazepine metabolites at Victoria Police and the Victorian Institute of Forensic Medicine. Table S2. Summary of the analytical detections in Victoria Police seizure data by number of cases and by number of tablets (March 2020–August 2022, n = 623 cases, n = 35 068 tablets) and in clinical data from the Emerging Drugs Network of Australia – Victoria (EDNAV) project (September 2020–August 2022, n = 125 cases). [file ADD-120-2465-s001.docx]

| **Supplementary Table 1.** List of monitored benzodiazepines and benzodiazepine metabolites at Victoria Police and the Victorian Institute of Forensic Medicine. | | |
| --- | --- | --- |
|  | **Victoria Police** | **Victorian Institute of Forensic Medicine** |
| **Prescription benzodiazepines** | | |
| 7-Aminoclonazepam | N^^^ | Y |
| 7-Aminoflunitrazepam | N^^^ | Y |
| 7-Aminonitrazepam | N^^^ | Y |
| Alprazolam | Y | Y |
| Chlordiazepoxide | Y | N |
| Clobazam | Y | Y |
| Clonazepam | Y | Y |
| Diazepam | Y | Y |
| Flunitrazepam | Y | Y |
| Lorazepam | Y | Y |
| Medazepam | Y | N |
| Midazolam | Y | Y |
| Nitrazepam | Y | Y |
| Nordiazepam | Y | Y |
| Oxazepam | Y | Y |
| Temazepam | Y | Y |
| **Benzodiazepine-type new psychoactive substances** | | |
| 4-Chlorodiazepam | N^^^ | Y |
| 3-Hydroxyphenazepam | Y | N |
| 8-Aminoclonazolam | Y | Y |
| Adinazolam | Y | Y |
| Bromazepam | Y | Y |
| Bromazolam | Y | Y |
| Chloro deschloroalprazolam | Y | N |
| Clobromazolam | Y | Y^†^ |
| Clonazolam | Y | Y |
| Delorazepam | N | Y |
| Desalkylflurazepam | Y^#^ | Y |
| Deschloroetizolam | Y | Y |
| Diclazepam | Y | Y |
| Estazolam | N | Y |
| Etizolam | Y | Y |
| Flualprazolam | Y | Y |
| Flubromazepam | Y | Y |
| Flubromazolam | Y | Y |
| Lormetazepam | N | Y |
| Meclonazepam | N | Y |
| Metizolam | N | Y |
| Nifoxipam | N | Y |
| Nimetazepam | Y | Y |
| Nitrazolam | N | Y |
| Phenazepam | Y | Y |
| Prazepam | N | Y |
| Pyrazolam | Y | Y |
| Triazolam | Y | N |
| ^^^ Detection of drug metabolites are not generally applicable to analysis of physical drug seizures but can be evident as breakdown products.  ^#^ Detected by the Drug Sciences Group outside of the study period.  ^†^ Formally added to the Victorian Institute of Forensic Medicine analytical toxicology screen in May 2022. | | |

| Supplementary Table 2. Summary of the analytical detections in Victoria Police seizure data by number of cases and by number of tablets (March 2020 – August 2022, n=623 cases, n=35,068 tablets) and in clinical data from the Emerging Drugs Network of Australia – Victoria (EDNAV) project (September 2020 – August 2022, n=125 cases). | | | |
| --- | --- | --- | --- |
|  | Police seizure data: number of cases n (%) | Police seizure data: number of tablets n (%) | EDNAV data:  number of cases n (%) |
| Total | **623** | **35,068** | **125** |
| Benzodiazepines (prescription and new psychoactive substances)^†^ | | | |
| Adinazolam | 16 (2.6) | 129 (0.4) | - |
| Alprazolam | 200 (32.1) | 9727 (27.7) | 19 (15.2) |
| Bromazolam | 9 (1.4) | 1843 (5.3) | 11 (8.8) |
| Clobromazolam (phenazolam) | 17 (2.7) | 423 (1.2) | 26 (20.8) |
| Chloro deschloroalprazolam | 1 (0.2) | 124 (0.4) | - |
| Clonazepam | 1 (0.2) | 3 (0) | 11 (8.8) |
| Clonazolam | 116 (18.6) | 5593 (15.9) | 49 (39.2) |
| *Aminoclonazolam* | *1 (0.2)* | *5 (0)* | *43 (34.4)* |
| Deschloroetizolam | 2 (0.3) | 466 (1.3) | 1 (0.8) |
| Diazepam | 3 (0.5) | 225 (0.6) | 38 (30.4) |
| Diclazepam | 1 (0.2) | 1 (0) | - |
| Estazolam | - | - | 2 (1.6) |
| Etizolam | 186 (29.9) | 16203 (46.2) | 29 (23.2) |
| Flualprazolam | 69 (11.1) | 1617 (4.6) | 24 (19.2) |
| Flubromazepam | 3 (0.5) | 6 (0) | 10 (8) |
| Flubromazolam | 19 (3) | 613 (1.7) | 2 (1.6) |
| Nitrazepam | - | - | 1 (0.8) |
| Phenazepam | - | - | 22 (17.6) |
| Other prescription medications | | | |
| Amantadine | 1 (0.2) | 10 (0) | - |
| Amitriptyline | - | - | 1 (0.8) |
| Amphetamine | - | - | 4 (3.2) |
| Aripiprazole | - | - | 1 (0.8) |
| Atropine | 1 (0.2) | 1 (0) | 1 (0.8) |
| Benzocaine | 2 (0.3) | 2 (0) | - |
| Buprenorphine | - | - | 3 (2.4) |
| Codeine | - | - | 13 (10.4) |
| Citalopram | - | - | 9 (7.2) |
| Cyproheptadine | 1 (0.2) | 10 (0) | 1 (0.8) |
| Diphenhydramine | 2 (0.3) | 9 (0) | 1 (0.8) |
| Doxepin | 7 (1.1) | 14 (0) | - |
| Doxylamine | - | - | 3 (2.4) |
| Duloxetine | - | - | 2 (1.6) |
| Fentanyl | - | - | 1 (0.8) |
| Fluoxetine | - | - | 3 (2.4) |
| Lamotrigine | - | - | 2 (1.6) |
| Levamisole | - | - | 2 (1.6) |
| Levetiracetam | - | - | 1 (0.8) |
| Lidocaine | 19 (3) | 808 (2.3) | 5 (4) |
| Mestanolone | 1 (0.2) | 2 (0) | - |
| Methadone | - | - | 16 (12.8) |
| Mirtazapine | - | - | 7 (5.6) |
| Moclobemide | - | - | 1 (0.8) |
| Modafinil | - | - | 1 (0.8) |
| Morphine | - | - | 2 (1.6) |
| Olanzapine | - | - | 3 (2.4) |
| Oxycodone | 1 (0.2) | 2 (0) | 7 (5.6) |
| Oxymetholone | 1 (0.2) | 2 (0) | - |
| Paliperidone | - | - | 3 (2.4) |
| Paroxetine | - | - | 2 (1.6) |
| Pregabalin | - | - | 2 (1.6) |
| Promethazine | 1 (0.2) | 10 (0) | 13 (10.4) |
| Quetiapine | 1 (0.2) | 1 (0) | 6 (4.8) |
| Sertraline | - | - | 2 (1.6) |
| Tramadol | - | - | 3 (2.4) |
| Venlafaxine | - | - | 1 (0.8) |
| Zopiclone | - | - | 1 (0.8) |
| Established illicit substances | | | |
| Cannabis (THC) | - | - | 30 (24) |
| Cocaine | - | - | 17 (13.6) |
| Heroin^‡^ | - | - | 11 (8.8) |
| Ketamine | 1 (0.2) | 12 (0) | 7 (5.6) |
| Lysergic acid diethylamide (LSD) | - | - | 1 (0.8) |
| Methylamphetamine^^^ | - | - | 70 (56) |
| 3,4-Methylenedioxymethamphetamine (MDMA)^*^ | - | - | 10 (8) |
| Other new psychoactive substances | | | |
| Desmethyltramadol | 2 (0.3) | 41 (0.1) | - |
| Etodesnitazene | 2 (0.3) | 41 (0.1) | - |
| Methylone | - | - | 1 (0.8) |
| N-ethylheptedrone | 2 (0.3) | 2 (0) | - |
| 4-Chloromethcathinone | - | - | 1 (0.8) |
| 5F-Cumyl-PINACA | - | - | 1 (0.8) |
| ^†^ Figures for alprazolam, clonazepam, diazepam and nitrazepam (benzodiazepines approved by the Therapeutic Goods Administration in Australia) for the EDNAV cases exclude where the benzodiazepine was a known prescription medication for an individual, where it was reported as an acute exposure proximate to hospital presentation or administered as part of pre-hospital or hospital management.  ^‡^ 6-Monoacetylmorphine was detected in n=2/11 of the EDNAV cases, the remaining cases involved the co-detection of codeine and morphine in addition to circumstantial evidence of heroin use proximate to patient presentation where codeine was not a regular medication, reported exposure or administered therapeutically.  ^^^ Amphetamine was co-detected with methylamphetamine in n=68/70 of the EDNAV cases.  ^*^ 3,4-Methylenedioxyamphetamine (MDA) was co-detected in n=8/10 of the EDNAV cases, nil cases where MDA was detected alone in the absence of MDMA. | | | |
